# ‘Trend in premature mortality from four major NCDs in Nanjing, China, 2007–2018’

**DOI:** 10.1186/s12889-021-12018-7

**Published:** 2021-11-25

**Authors:** Huafeng Yang, Yali Fu, Xin Hong, Hao Yu, Weiwei Wang, Fengxia Sun, Jinyi Zhou, Nan Zhou

**Affiliations:** 1 Nanjing Municipal Center for Disease Control and Prevention, Nanjing, Jiangsu China; 2 Jiangsu Health Development Research Center, Nanjing, Jiangsu China; 3 Jiangsu Provincial Center for Disease Control and Prevention, Nanjing, Jiangsu China

**Keywords:** Non-communicable diseases (NCDs), Premature mortality, Average annual percentage changes (AAPC), Trend

## Abstract

**Background:**

This study aims to analyze the trends of premature mortality caused from four major non-communicable diseases (NCDs), namely cardiovascular disease (CVD), cancer, chronic respiratory diseases, and diabetes in Nanjing between 2007 and 2018 and project the ability to achieve the “Healthy China 2030” reduction target.

**Methods:**

Mortality data of four major NCDs for the period 2007–2018 were extracted from the Death Information Registration and Management System of Chinese Center for Disease Control and Prevention. Population data for Nanjing were provided by the Nanjing Bureau of Public Security. The premature mortality was calculated using the life table method. Joinpoint regression model was used to estimate the average annual percent changes (AAPC) in mortality trends.

**Results:**

From 2007 to 2018, the premature mortality from four major NCDs combined in Nanjing decreased from 15.5 to 9.5%, with the AAPC value at − 4.3% (95% CI [− 5.2% to − 3.4%]). Overall, it can potentially achieve the target, with a relative reduction 28.6%. The premature mortality from cancer, CVD, chronic respiratory diseases and diabetes all decreased, with AAPC values at − 4.2, − 5.0%, − 5.9% and − 1.6% respectively. A relative reduction of 40.6 and 41.2% in females and in rural areas, but only 21.0 and 12.8% in males and in urban areas were projected.

**Conclusion:**

An integrated approach should be taken focusing on the modifiable risk factors across different sectors and disciplines in Nanjing. The prevention and treatment of cancers, diabetes, male and rural areas NCDs should be enhanced.

## Background

Non-communicable diseases (NCDs) are becoming the leading cause of death worldwide, and considered as the major health challenges in the twenty-first century [1]. In 2016, NCDs collectively caused 41 million deaths worldwide, equivalent to 71% of all global deaths. Cardiovascular disease (CVD), cancers, chronic respiratory diseases and diabetes attribute to 80% of NCDs related deaths [2]. In China, death from non-communicable disease in 2016 accounted for 89% of all deaths, of them 77% were due to these four major NCDs [1]. Increased non-communicable disease burden would lead to a shortage of health resources, increased treatment costs and delayed economic growth. It was estimated that the risk of a 30-years-old person dying from any of four major NCDs before reaching the age of 70 years was 17% in China. This is lower than the global risk (18%), and with a slightly higher risk for males (20%) than for females (14%).

World Health Organization (WHO) recognizes premature mortality (defined as the probability of dying between the ages of 30 years and 70 years) as an important indicator in assessing the level of NCDs prevention and control in a region that is not affected by age composition [3–5]. In 2012, WHO proposes to reduce premature mortality from four major NCDs by 25% relative to 2010 levels by 2025 [6, 7]. The United Nation’s Sustainable Development Goals for 2030 includes the aim of reducing premature mortality from NCDs by one third (relative to 2015 levels) [8], while “Healthy China 2030” proposes to reduce premature mortality by 10% by 2020 and 30% by 2030 [9]. Previous studies suggested that there were significant different in premature mortality caused by four major NCDs and their change speed among provinces and the task of achieving “Healthy China 2030” reduction target would be daunting [10–13]. However, they were gender and geographic alone studies or used the annual growth rate only. However, whether their findings can be generalized to other cities in China is unknown.

Nanjing, the provincial capital city of eastern China Jiangsu Province, is one of the important researches and education bases and a critical transportation hub in the country, with a population of 8.5 million at the end of 2018. Previous studies reported that the top 3 causes of death in Nanjing were non-communicable diseases, including CVD, cancers and chronic respiratory diseases [14]. To facilitate policy makers to implement preventative strategies and achieving “Healthy China 2030” target, the present study aimed to evaluate the trends in premature mortality from four major NCDs in Nanjing in the last decade, focusing on the gender and geographic difference.

## Methods

### Data collection

Mortality data of four major NCDs from 2007 to 2018 in Nanjing were extracted from the Death Information Registration and Management System which is operated by Chinese Center for Disease Control and Prevention (CDC). Household registration population data were provided by the Nanjing Bureau of Public Security.

The death information registry, implemented since 2007, records in detail of the death information, including sex, date of birth, date of death, underlying causes. All categories of causes of death are coded using the International Classification of Diseases 10th Edition (ICD-10) [15]. Four major NCDs were identified and classified according to the death cause statistics section of the WHO Global Health Report, including cardiovascular disease [ICD-10: I00-I99], cancers [ICD-10: C00-C97], chronic respiratory diseases [ICD-10: J30-J98] and diabetes [ICD-10: E10-E14]. The data were subject to the three-level quality control of medical institutions, district CDC and municipal CDC, and reviewed monthly with public security, civil affairs and other departments to ensure the accuracy of the data [16].

### Statistical analysis

The primary indicator of this study was premature mortality from four major NCDs whereas the second indicator was age-standardized premature mortality rates (ASPMR). Using the direct standardization method, ASPMR of four major NCDs were calculated as number of deaths per 100,000 residents by age groups, based on the 2000 China’s Fifth Census Data.

Referring to WHO’s definition, premature mortality was considered as death of 30–70 years old (excluding 70 years old). Using the life table method, the premature mortality between the exact ages of 30 and 70, from any of the four causes and in the absence of other causes of death, was calculated using the equations below [17].

Mortality rates according to five-year age groups (_5_^*^M_x_) were first calculated:

$$ {5}^{\ast }{\mathrm{M}}_x=\frac{\mathrm{Total}\ \mathrm{deaths}\ \mathrm{from}\ \mathrm{four}\ \mathrm{NCD}\ \mathrm{causes}\ \mathrm{between}\ \mathrm{exact}\ \mathrm{age}\ \mathrm{x}\ \mathrm{and}\ \mathrm{exact}\ \mathrm{age}\ \mathrm{x}+5}{\mathrm{Total}\ \mathrm{population}\ \mathrm{between}\ \mathrm{exact}\ \mathrm{age}\ \mathrm{x}\ \mathrm{and}\ \mathrm{exact}\ \mathrm{age}\ \mathrm{x}+5} $$

For each five-year age group, the probability of mortality from four major NCDs (_5_^*^q_x_) was calculated using the following formula:

$$ {{}_5{}^{\ast }q}_{\mathrm{x}}=\frac{{{}_5{}^{\ast}\mathrm{M}}_{\mathrm{x}}\ast 5}{1+{{}_5{}^{\ast}\mathrm{M}}_{\mathrm{x}}\ast 2.5} $$

The unconditional probability of death, for the 30–70 age range, was calculated last:

$$ {{}_{40}{}^{\ast }q}_{30}=1-\prod \limits_{x=30}^{65}\left(1-{{}_5{}^{\ast }q}_x\right) $$

The changes in mortality time trend were described using joinpoint regression analysis. Permutation test was used to determine the statistically significant joinpoint points in the model. According to the requirements of the model, at most 2 joinpoints can be selected for 12 data points. We reported the best model recommended by the Joinpoint Regression Program. To quantify the trend over the whole period, the average annual percent change (AAPC) and corresponding 95% confidence interval (CI) were evaluated [18]. AAPC was computed as a geometric weighted average of various annual percent change (APC) values from the regression analysis. We projected ASPRM and premature mortality from four major NCDs for 2030 by fitting non-linear analysis model by the joinpoint regression, and the formula is as follows [19].

$$ E\kern0.10em \left[\kern0.10em {y}_i\kern0.28em |\kern0.28em {x}_i\right]={e}^{\beta_0+{\beta}_1{x}_i+{\delta}_1{\left({x}_i-{\Gamma}_1\right)}^{+}+\cdots +{\delta}_1{\left({x}_i-{\Gamma}_k\right)}^{+}} $$

Microsoft Excel (version 2019) and Joinpoint Regression Program (Version 4.7.0.0) were used for this study. APC > 0 means that the rate has increased annually in a certain period of time, APC < 0 means that the rate has decreased annually in a certain period. If there is no joinpoint, then APC = AAPC, which means the rate fluctuated during the total study period. A two-sided *P* value of less than 0.05 was considered statistically significant.

## Result

Over the 12-year study period from 2007 to 2018, a total of 105,761 premature deaths of four major NCDs were recorded in Nanjing. Cancers were found to be the most common causes among those four major NCDs (64,996, 61.5% of the total), followed by CVD (34,128, 32.3%), chronic respiratory diseases (3328, 3.2%) and diabetes (3309, 3.1%). The number of deaths in males were almost twice than in females (70,695 vs. 35,066, M/F: 2.0), but 20% lower in urban areas than in rural areas (45,514 vs. 60,247, U/R: 0.8).

**Fig. 1:**
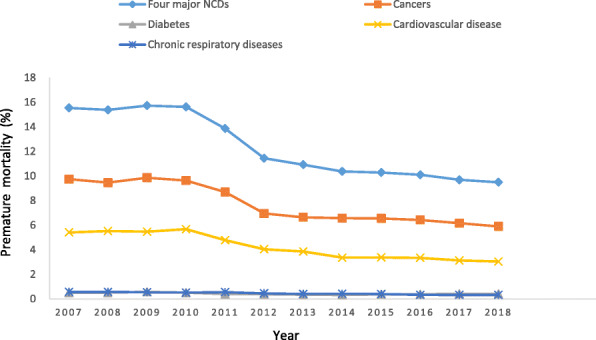
The premature mortality from the four major NCDs in Nanjing, 2007–2018

As shown in Fig. 1, continuing declining in the age-standardized premature mortality rates and probability of premature mortality were observed. ASPMR changed from 265.6 per 100,000 people in 2007 to 155.6 per 100,000 people in 2018 (Table 1). Joinpoint analysis showed a significant decrease in ASPMR, with AAPC of − 4.6% (95% confidence interval [CI], − 6.0% to − 3.2%), and identified three patterns. Specifically, it slightly decreased from 2007 to 2010 (APC − 0.5%) before a sharp decreasing from 2007 to 2013 (APC − 12.1%) and 2013 to 2018 (APC − 2.3%) (Table 3). The premature mortality decreased from 15.5% in 2007 to 9.5% in 2018 (Table 1, Fig. 1). For premature mortality, joinpoint analysis showed a significant decrease, with AAPC of − 4.3% (95% CI, − 5.3% to − 3.4%). From 2007 to 2010, there was a non-significant decrease of 0.07%. From 2010 onwards, the probability significantly decreased, with APC of − 11.9%, followed by another significantly decreasing trend of − 2.2%. (Table 4).

**Table 1:** Age-standardized premature mortality rates (per 100,000) and premature mortality (%) of four major NCDs and its subcategory in Nanjing, 2007–2018

Year	Four major NCDs		Cancers		Diabetes		Cardiovascular disease		Chronic respiratory diseases	
	ASPMR(95%CI)	premature mortality	ASPMR(95%CI)	premature mortality	ASPMR(95%CI)	premature mortality	ASPMR(95%CI)	premature mortality	ASPMR(95%CI)	premature mortality
2007	265.6 (265.2–266.0)	15.5	164.2 (163.9–164.5)	9.7	7.2 (7.2–7.3)	0.5	85.9 (85.7–86.1)	5.4	8.3 (8.2–8.3)	0.6
2008	262.1 (261.7–262.5)	15.4	158.4 (158.1–158.7)	9.5	7.8 (7.7–7.8)	0.5	87.7 (87.5–88.0)	5.5	8.2 (8.1–8.3)	0.6
2009	267.5 (267.1–268.0)	15.7	164.7 (164.3–165.0)	9.9	8.4 (8.4–8.5)	0.6	86.6 (86.4–86.8)	5.5	7.9 (7.8–8.0)	0.5
2010	262.8 (262.4–263.2)	15.6	159.4 (159.1–159.7)	9.6	7.6 (7.6–7.7)	0.5	88.6 (88.3–88.8)	5.7	7.2 (7.2–7.3)	0.5
2011	230.9 (230.5–231.3)	13.9	141.9 (141.6–142.2)	8.7	5.7 (5.7–5.8)	0.4	75.4 (75.2–75.6)	4.8	7.8 (7.7–7.9)	0.6
2012	192.3 (192.0–192.7)	11.4	115.3 (115.0–115.6)	7.0	5.6 (5.6–5.7)	0.4	64.8 (64.6–65.0)	4.0	6.6 (6.5–6.7)	0.5
2013	181.7 (181.3–182.0)	10.9	108.8 (108.6–109.1)	6.6	5.3 (5.3–5.4)	0.4	61.6 (61.4–61.8)	3.9	5.9 (5.8–5.9)	0.4
2014	171.9 (171.6–172.2)	10.4	107.1 (106.9–107.4)	6.6	4.8 (4.7–4.8)	0.3	53.8 (53.6–53.9)	3.4	6.2 (6.2–6.3)	0.4
2015	172.0 (171.7–172.3)	10.3	107.1 (106.8–107.4)	6.6	5.5 (5.4–5.5)	0.4	53.8 (53.7–54.0)	3.4	5.6 (5.5–5.6)	0.4
2016	168.9 (168.6–169.3)	10.1	104.7 (104.4–104.9)	6.4	5.6 (5.5–5.6)	0.4	53.8 (53.6–53.9)	3.3	4.9 (4.9–5.0)	0.3
2017	161.2 (160.9–161.6)	9.7	100.1 (99.9–100.4)	6.2	6.2 (6.2–6.3)	0.4	50.2 (50.0–50.4)	3.1	4.7 (4.7–4.8)	0.3
2018	155.6 (155.2–155.9)	9.5	96.0 (95.8–96.29)	5.9	6.2 (6.1–6.3)	0.4	48.8 (48.6–48.3)	3.0	4.6 (4.5–4.6)	0.3

Abbreviations: ASPMR, Age-standardized premature mortality rates; NCDs, Non-communicable diseases

### Trend in premature mortality of four major NCDs in different subcategories

From 2007 to 2018, the ASPMR from four major NCDs showed a downward trend annually (Table 1). Joinpoint regression analysis indicated that the ASPMR decrease significantly in cancers, CVD and chronic respiratory diseases, with the AAPC of − 4.5, − 5.0% and − 5.7%, respectively, showing significant changes, without detectable joinpoint in chronic respiratory diseases (Table 3 and Figure1). Trends of premature mortality were similarly illustrating continuously downward trends in cancers, CVD and chronic respiratory diseases with AAPC of − 4.2, − 5.0% and − 5.9%, respectively (Table 4). For diabetes, the ASPMR continuously increased until 2009 (APC 7.1%), then, a decreasing trend from 2009 to 2013 (APC − 12.3%), whereas an increasing trend from 2013 to 2018 (APC 5.1%) was observed without significance (AAPC -1.3, 95% CI [− 6.1 to 3.8%]) (Table 3). Similarly, the premature mortality of diabetes remained relatively stable over the period (AAPC -1.6, 95% CI [− 6.7 to 3.8%]) (Table 4).

### Trend in premature mortality of four major NCDs with different genders

**Fig. 2:**
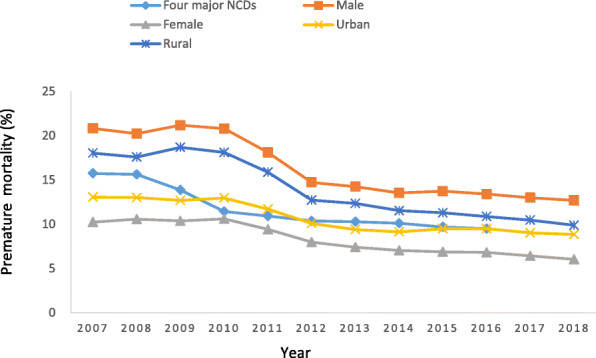
The premature mortality from the four major NCDs by gender and geographic in Nanjing, 2007–2018

From 2007 to 2018, the ASPMR in both males and females showed a downtrend annually, decreasing from 362.3 per 100,000 people to 210.0 per 100,000 people, and from 173.0 per 100,000 people to 101.0 per 100,000 people, respectively (Table 2). Remarkably decreased trends of ASPMR of four major NCDs in both males (AAPC − 4.7%) and females (AAPC − 4.7%) were observed (Table 3). For premature mortality, the declines were slightly larger in males (20.8 to 12.7%) than in females (10.2 to 6.0%) (Table 2 and Fig. 2). While a significant decrease in the premature mortalities were observed in both genders during the study period (AAPC − 4.2% for males and − 4.6% for females, respectively), with three trends. During 2010 to 2013, the premature mortality in both males and females significantly decreased with AAPC of − 12.5% and − 11.4% (Table 4).

**Table 2:** Age-standardized premature mortality rates (per 100,000) and premature mortality (%) of four major NCDs by gender and geographic in Nanjing, 2007–2018

Year	Male		Female		Urban		Rural	
	ASPMR(95%CI)	premature mortality	ASPMR(95%CI)	premature mortality	ASPMR(95%CI)	premature mortality	ASPMR(95%CI)	premature mortality
2007	362.3 (361.6–362.9)	20.8	173.0 (172.5–173.4)	10.2	209.9 (209.3–210.5)	13.1	321.1 (320.5–321.6)	18.0
2008	350.7 (350.1–351.4)	20.2	177.5 (177.0–177.9)	10.6	206.7 (206.1–207.3)	13.0	313.5 (313.0–314.1)	17.6
2009	366.0 (365.3–366.6)	21.2	175.1 (174.6–175.6)	10.4	202.5 (201.9–203.1)	12.7	331.5 (330.9–332.1)	18.7
2010	358.0 (357.4–358.7)	20.8	174.3 (173.9–174.8)	10.6	203.1 (202.5–203.7)	13.0	319.1 (318.5–319.7)	18.1
2011	304.3 (303.7–304.9)	18.1	156.6 (156.1–157.0)	9.4	183.5 (182.9–184.0)	11.7	274.6 (274.1–275.1)	15.9
2012	250.7 (250.2–251.3)	14.7	132.3 (131.9–132.7)	8.0	161.4 (160.9–162.0)	10.1	220.4 (219.9–220.8)	12.7
2013	239.7 (239.1–240.2)	14.2	122.2 (121.8–122.6)	7.4	149.2 (148.7–149.7)	9.4	211.5 (211.0–211.9)	12.3
2014	226.5 (226.0–227.1)	13.5	115.9 (115.5–116.3)	7.0	144.2 (143.7–144.7)	9.1	197.1 (196.7–197.6)	11.5
2015	229.2 (228.7–229.8)	13.7	113.7 (113.3–114.1)	6.9	148.9 (148.4–149.4)	9.5	192.5 (192.1–193.0)	11.3
2016	224.0 (223.4–224.5)	13.4	113.2 (112.8–113.5)	6.8	149.9 (149.4–150.4)	9.5	185.4 (185.0–185.8)	10.9
2017	214.7 (214.1–215.2)	13.0	107.3 (106.9–107.7)	6.4	142.4 (141.9–142.9)	9.0	177.2 (176.7–177.6)	10.5
2018	209.9 (209.4–210.4)	12.7	101.0 (100.6–101.4)	6.0	138.8 (138.3–139.3)	8.9	168.3 (167.9–168.7)	9.9

Abbreviations: ASPMR, Age-standardized premature mortality rates; NCDs, Non-communicable diseases

**Table 3:** Joinpoint analysis of trends in age-standardized premature mortality rates (ASPMR) of four major NCDs in Nanjing (2007–2018)

	Total Study Period	Trend 1		Trend 2		Trend 3	
	AAPC (%, 95 CI)	years	APC (%)	years	APC (%)	years	APC (%)
All	−4.6*(−6.0 ~ −3.2)	2007–2010	−0.5	2010–2013	−12.1*	2013–2018	−2.3*
Subcategory							
Cancers	−4.5*(−6.3 ~ −2.7)	2007–2010	− 0.9	2010–2013	− 12.1*	2013–2018	− 1.9*
Diabetes	− 1.3(− 6.1 ~ 3.8)	2007–2009	7.1	2009–2013	− 12.3*	2013–2018	5.1
Cardiovascular disease	−5.0*(− 6.7 ~ − 3.2)	2007–2010	− 0.3	2010–2014	− 10.9*	2014–2018	− 2.2
Chronic respiratory diseases	−5.7*(− 6.7 ~ − 4.8)	2007–2018					
Gender							
Male	−4.7*(− 6.5 ~ − 2.9)	2007–2010	− 0.7	2010–2013	− 13.0*	2013–2018	− 1.8
Female	−4.7*(− 6.4 ~ − 2.9)	2007–2010	0.0	2010–2013	− 11.6*	2013–2018	− 3.1*
Geographic							
Urban	−3.5*(− 5.6 ~ − 1.3)	2007–2010	− 1.3	2010–2013	−9.6*	2013–2018	−1.0
Rural	− 5.6*(−7.1 ~ − 4.0)	2007–2010	− 0.3	2010–2013	−13.7*	2013–2018	− 3.5*

Abbreviations: AAPC, the average annual percent change; APC, the annual percent change. * indicates that AAPC or APC significantly different from 0 (two-sided *p* < 0.05)

**Table 4:** Joinpoint analysis of trends in the premature mortality of four major NCDs in Nanjing (2007–2018)

	Total Study Period	Trend 1		Trend 2		Trend 3	
	AAPC (%, 95 CI)	years	APC (%)	years	APC (%)	years	APC (%)
All	− 4.3*(− 5.3 ~ − 3.4)	2007–2010	0.1	2010–2013	−11.9*	2013–2018	− 2.2*
Subcategory							
Cancers	−4.2*(− 5.9 ~ − 2.5)	2007–2010	− 0.2	2010–2013	− 11.8*	2013–2018	−1.7
Diabetes	−1.6(− 6.7 ~ 3.8)	2007–2009	6.3	2009–2013	− 12.4	2013–2018	4.7
Cardiovascular disease	− 5.0*(− 6.9 ~ − 3.2)	2007–2010	0.1	2010–2014	−11.4*	2014–2018	−2.2
Chronic respiratory diseases	−5.9*(− 6.9 ~ − 4.8)	2007–2018					
Gender							
Male	− 4.2*(− 6.0 ~ −2.5)	2007–2010	0.2	2010–2013	− 12.5*	2013–2018	− 1.4
Female	−4.6*(− 6.4 ~ − 2.7)	2007–2010	0.7	2010–2013	− 11.4*	2013–2018	− 3.4*
Geographic							
Urban	−3.3*(−5.4 ~ −1.1)	2007–2010	− 0.5	2010–2013	−9.9*	2013–2018	−0.7
Rural	− 5.2*(− 6.8 ~ − 3.4)	2007–2010	0.2	2010–2013	− 12.9*	2013–2018	− 3.4*

Abbreviations: AAPC, the average annual percent change; APC, the annual percent change. * indicates that AAPC or APC significantly different from 0 (two-sided *p* < 0.05)

### Trend in premature mortality of four major NCDs with different geographics

A downtrend in ASPMR in both urban and rural areas was observed. The ASPMR decreased by 3.5% from 209.9 to 138.8 per 100,000 people in urban areas, and decreased by 5.6% from 321.1 to 168.3 per 100,000 people in rural areas (Tables 2, 3). Joinpoint analysis showed a significantly decreasing trend of premature mortality by 3.3% per year in urban areas. As in rural areas, it decreased with the AAPC of − 5.2% (Table 4).

### Prediction and analysis of joinpoint regression model

Table 5 presents the prediction of the premature mortality from four major NCDs to reach the “Healthy China 2030” target in Nanjing. It is possible to achieve the target from four major NCDs combined, with the relative reduction 28.6%. Among the subcategories, diabetes is the least likely to achieve the target, which even showed a 94.2% increase. Another disease failing to meet the target would be cancer, with a 24.7% relative reduction. Both CVD and chronic respiratory diseases shared a more than 30% relative reduction in premature mortality, with the latter showing the greater reduction at 61.0%.

**Table 5:** Observed AMPMR and premature mortality of four major NCDs in 2015 and predicted values for 2030 in Nanjing (2007–2018)

	AMPMR			premature mortality		
	Observed 2015	Predicted 2030	Relative reduction (%)	Observed 2015	Predicted 2030	Relative reduction (%)
All	172	118.8	−30.9	10.3	7.3	− 28.6
Subcategory						
Cancers	107.1	78.5	−26.7	6.6	4.9	−24.7
Diabetes	5.5	11.4	107.1	0.4	0.7	94.2
Cardiovascular disease	53.8	37.9	−29.7	3.4	2.4	−30.3
Chronic respiratory diseases	5.6	2.3	−59.5	0.4	0.2	−61.0
Gender						
Male	229.2	171.7	−25.1	13.7	10.9	−21.0
Female	113.7	70.8	−37.7	6.9	4.1	−40.6
Geographic						
Urban	148.9	126.3	−15.2	9.5	8.3	−12.8
Rural	192.5	110.7	−42.5	11.3	6.6	−41.2

PS: Relative reduction (%) = (projected premature mortality 2030-observed premature mortality 2015) / (observed premature mortality 2015)*100%

A difference was seen in the possibility to reach the target from four major NCDs for both gender and geographic. A relative reduction of 40.6% in females was projected, but only 21.0% in males. In urban areas, a slightly smaller reduction (12.8%) was projected compared to a greater than 30% reduction in rural areas.

## Discussion

This study confirmed a significant downward trend on both ASPMR and premature mortality from four major NCDs combined from 2007 to 2018 in Nanjing, with a rapid declining since 2010. This finding was in line with the global trend (22 to 18%) and national trend (30.7 to 18%) scenarios [4, 13]. Previously published data indicated that the premature mortality of four major NCDs in Nanjing in 2015 was 10.3%, which was lower than the nation level (18.5%), Jiangsu Province (13.4%), Beijing (11.1%), Tianjin (12.9%), and Chongqing (16.0%, in 2016), but higher than Shanghai (8.4%). It was also comparable to the level of developed countries [1, 10, 11, 13, 20]. In this study, we found that premature mortality from NCDs in Nanjing has continuously declined since 2007. If this decline trend continues, it will be possible to reach the “Healthy China 2030” target.

The premature mortalities from four major NCDs in Nanjing in 2015 respectively, were all lower than the levels of national and some other regional [20–24]. The premature mortalities of four major NCDs in western regions are higher than the eastern regions in China, and they are lower in the economically developed regions. Our results showed that the declines of the premature mortality rates from four major NCDs rank in following order: chronic respiratory diseases (AAPC -5.9%), CVD (AAPC − 5.0%), cancer (AAPC − 4.2%), and diabetes (AAPC − 1.6%), which is consistent with the national trend [13]. In general, the chronic respiratory diseases and CVD may reach the 2030 target by reducing the premature mortality rates by 30.3 and 61.0%, however, there would be 5.3% short of the target for cancer. In addition, it seems unlikely to reach the target for diabetes.

Inconsistent with the national data, our study indicated that the greatest contributor to premature mortality was cancer [13]. Multiple factors can contribute occurrence and development of malignant tumors, among them smoking and chronic infections are considered as the most important risk factors [25, 26]. Previous studies reported that the top 5 mortality rates caused by tumor in Nanjing were similar to the Jiangsu Province and the national data, with slight differences in order. The mortality rate of lung cancer ranks first, mainly due to tobacco smoking behavior, air pollution and other environmental factors including decoration, cooking oil fume [27].

In line with the global and the national level, CVD ranks first in mortality rates among all the deaths in Nanjing. The control of hypertension has a great impact on the reduction of premature cardiovascular death in the world. Smoking control was reported as an efficient way to reduction of early cardiovascular death in males, whilst obesity control can be critical to reduce early cardiovascular death in females [28].

For diabetes, joinpoint analysis showed a non-significant decrease in the premature mortality. It might be explained by the elevated incidence of diabetes [29]. In Jiangsu Province, mortality rate of diabetes is at a lower level among people younger than 55 years old, and it increases significantly after 55 [30]. Accompanied by lifestyle changes, long duration of sedentary behavior, unhealthy diet and obesity were all considered as risk factors to diabetes. Furthermore, diabetes increased the risk of CVD and premature death [31].

In general, chronic respiratory diseases mainly include tuberculosis, diffuse pulmonary fibrosis, chronic obstructive pulmonary disease (COPD), bronchiectasis, and bronchial asthma. The incidence, prevalence and the mortality of COPD in Jiangsu Province were higher than the global average [32, 33]. Genetic susceptibility, smoking, air pollution, occupational exposure, infection, socioeconomic conditions are all recognized risk factors for COPD [34].

Furthermore, the ASPMR of four major NCDs showed a downward trend in both males and females aged 30 to 70 during the study period. The premature mortality of males was about twice that of females, and the gap was consistent with that of Beijing and the national data [10, 13], but higher than that of the global average level [35]. The monitoring report of NCDs in Nanjing in 2017 showed the rates of smoking (37.2%), drinking (47.7%), obesity (11.1%), hypertension (28.7%) and diabetes mellitus (10.30%) in males were higher than those in females. It might explain the gender differences in premature mortality rates. The prediction suggested that by 2030, the premature mortality in females can reach the target, while that in males still have a 9% gap from the target.

The ASPMR of four major NCDs showed downward trends in urban and rural areas. The trend of premature mortality in rural areas was consistent with that in urban areas, but the decline range of probability was higher than that in urban areas. The prediction suggested that by 2030, the premature mortality in rural areas may reach the target, while that in urban areas may not yet reached. The higher mortality rates in rural areas might be due to lower education and income levels, tobacco or unhealthy habits and lack access to medical and health care services [36]. The development of non-communicable diseases can be influenced by social, environmental, behavioral, nutritional and clinical factors [37]. Therefore, to prevent premature mortality from four major NCDs, active interventions to major risk factors are needed such as early prevention, early detection and early treatment through health promotion and related programs.

It is widely acknowledged that early prevention is important to prevent and control the NCDs. Interventions should focus on risk factors that are modifiable, namely tobacco smoking, alcohol drinking, excessive salt intake, obesity, elevated blood pressure and elevated blood sugar [17]. Tobacco Surveillance in Nanjing showed that the current smoking rate among local residents over 15 years old was 23.8% in average, but one in two males were smokers. In addition, the smoking rate of people at 15–24 years old in China was significantly higher than that of other age groups [38]. Therefore, it is important to enhance the enforcement of existing tobacco control regulations, increase tobacco and alcohol taxes, and improve the ability of medical and health institutions to provide smoking cessation assistance to people desired to quit smoking. Furthermore, interventions can also be promoting healthy diet, providing supportive environment such as healthy theme parks, public sports facilities, and strengthening environmental protection and supervision. In order to reduce the prevalence of related chronic infections in the population, increasing vaccination against human papillomavirus (HPV) and hepatitis B virus (HBV) among adolescents and general population can be effective. Medical and health care institutions should be encouraged to carry out public health education and health promotion at the population level. At the same time, more researches are needed to eliminate or reduce potential risk factors that affect patients’ morbidity and death.

Early detection including establishing a screening and early diagnosis mechanism, and standardizing the treatment of early cases are urgently needed. Early lung cancer screening advocates the application of low-dose spiral CT, which can reduce the mortality rate of early lung cancer to 20% [39]. Moreover, we can learn from Japanese and Korean regarding using early gastric cancer screening technology to improve the 5-year relative survival rate for patients [40, 41]. Some early diagnosis and treatment of gastrointestinal cancer and other mature screening technologies is currently carrying out in high-risk groups in Jiangsu Province. However, limited financial funds, low-level screening technology, and poor awareness of screening among the masses are noted.

In order to substantially reduce CVD and respiratory disease mortality, early treatment is also required at first-level (e.g. district), regional, and specialist hospitals including appropriate and effective referrals and high-quality long-term care, for acute cardiovascular disease, acute exacerbations of asthma and chronic obstructive pulmonary disease, and acute complications of diabetes.

### Limitation

According to the existing standards [16], the death cause monitoring data in Nanjing are real-time and reliable. However, this study was limited by a lack of comparison groups from other districts and counties. In addition, we didn’t perform separate analysis of the four types NCDs in males and females or in urban and rural areas. This may prevent us to a deep understanding of the differences in mortality. Another limitation was that although the data were subject to the three-level quality control, there might still be unavoidable underreporting and misclassification of diseases.

## Conclusion

Our study indicated that the “Healthy China 2030” target of reducing the probability of premature mortality by 30% due to four major NCDs in Nanjing can potentially be achieved. Despite this, the total number of premature mortalities from NCDs is still large. For these regions with a low probability of early death and a rapid decline in the past, it may be challenging maintain the decline trend to achieve the target. Unhealthy lifestyle are still highly prevalent in the population despite the behavior change efforts made by public health practitioners to reduce such risk exposures in the past decades [42, 43]. To achieve further decline in the premature mortality, an integrated approach should be taken focusing on the modifiable risk factors across different sectors and disciplines in Nanjing. The out control of those risk factors will lead to an increased disease burden of NCDs [44]. Prevention and treatment of cancers and diabetes may require additional efforts, especially scientific researches, optimization, and promotion of appropriate technologies for early diagnosis and treatment, as well as strengthening pre-hospital first aid. The health management should be prioritized to key populations such as males and people in rural areas.

## Data Availability

The datasets used in the current study are available from the corresponding authors on reasonable request.

## Abbreviations

NCDs: Non-communicable diseases
CVD: cardiovascular disease
AAPC: Average annual percentage changes
WHO: World Health Organization
ICD-10: International Classification of Diseases 10th Edition
CDC: Center for Disease Control and Prevention
ASPMR: Age-standardized premature mortality rates
CI: Confidence interval
APC: Annual percent change
COPD: Chronic obstructive pulmonary disorder
HPV: Human papillomavirus
HBV: Hepatitis B virus
CT: Computed tomography
